# Pregnancy outcomes in patients complicated with pre-excitation syndrome

**DOI:** 10.1007/s00404-024-07420-6

**Published:** 2024-03-02

**Authors:** Kana Wang, Junguo Xin, Qing Hu, Xiaodong Wang, Haiyan Yu

**Affiliations:** 1 Department of Obstetrics and Gynecology, West China Second University Hospital, Sichuan University, No. 20, 3rd Section, South Renmin Road, Chengdu, 610041 China; 2 Key Laboratory of Birth Defects and Related Diseases of Women and Children (Sichuan University), Ministry of Education, No. 20, 3rd Section, South Renmin Road, Chengdu, 610041 China; 3 School of Public Heath, Chengdu Medical College, No. 20, 3rd Section, South Renmin Road, Chengdu, 610041 China

**Keywords:** Gestation, Pre-excitation syndrome, Cardiac arrhythmia, Supraventricular tachycardia

## Abstract

**Introduction:**

Pregnant women with pre-excitation syndrome are more likely to develop supraventricular tachycardia (SVT) during pregnancy and delivery, leading to an increased risk of adverse events.

**Method:**

This was a retrospective study of 309 pregnancies in 280 women (29 women had two pregnancies in this series) with pre-excitation syndrome who delivered at West China Second University Hospital from June 2011 to October 2021. All the 309 pregnant women with pre-excitation syndrome were divided into SVT and non-SVT groups to analyze the cardiac and obstetric complications.

**Results:**

Among the included pregnant women in the past 10 years, the prevalence of pre-excitation syndrome was 0.24% (309/127725). There were 309 cases with pre-excitation syndrome in all hospitalized pregnant women. Among them, 62 (20.1%, 62/309) had a history of SVT. In the 62 cases with SVT during pregnancy, 22 (35.5%) cases had a history of SVT. Gestational diabetes mellitus was associated with SVT during pregnancy. The cesarean section rate was 88.7% in the SVT group, which was significantly higher than that in the non-SVT group (64.8%) (*P* < 0.001). Cases with SVT during pregnancy had more cardiac and obstetric complications. Four fetal deaths were recorded in the SVT group. Additionally, 29 women experienced two pregnancies during the study period, among whom, five received radiofrequency ablation after the first delivery and obtained better outcomes in the second pregnancy.

**Conclusion:**

The adverse outcomes such as cardiac complications, maternal and fetal complications (PROM, prematurity, SGA, fetal distress, etc.) in pregnant women with pre-excitation syndrome were closely related to SVT, with possible risk factors including history of SVT before pregnancy, cardiac function, heart organic abnormalities, and gestational diabetes mellitus.

## What does this study add to the clinical work

**Table:** 

Pre-excitation syndrome may result in life-threatening arrhythmias such as supraventricular tachycardia during pregnancy and delivery. This study was conducted to determine the correlation between supraventricular tachycardia and adverse maternal and fetal outcomes in patients with pre-excitation syndrome, and to find the risk factors leading to supraventricular tachycardia during pregnancy.	

## Introduction

Pre-excitation syndrome is a congenital defect of the cardiac conduction system defined as “a premature activation of the ventricular myocardium by an impulse that travels by an anomalous path” [1–3]. Pre-excitation syndrome can be mainly divided into Wolff–Parkinson–White syndrome (WPW), Lown-Ganong-Levine syndrome (LGL) and Mahaim-Type pre-excitation [1]. Kent bundle is the most common atrioventricular accessory pathway and Mahaim fiber is relatively rare [4]. As the most common form of pre-excitation, the WPW syndrome is commonly manifested as a short PR interval and prolonged QRS duration with an initial slurring upstroke on the electrocardiogram (ECG) due to early ventricular activation [5]. Hallmark electrocardiographic findings of LGL include a short PR interval, normal QRS complex, and paroxysmal tachycardia [6]. And other pre-excitation abnormalities associated with different accessory pathways are the Mahaim Fiber, named Mahaim-Type pre-excitation [3, 7]. Additionally, asymptomatic Wolff–Parkinson–White Syndrome, which is only detected on the electrocardiogram without any clinical symptoms, has been observed recently. Moreover, various subtypes of ventricular pre-excitation variants have also been identified, including the atriofascicular pathway, long and short decrementally conducting atrioventricular pathways, fasciculoventricular pathway, atrio-Hisian bypass tract, as well as nodoventricular and nodofascicular fibers [8, 9].

The clinical symptoms associated with the above ECG findings include tachycardia palpitations, episodic lightheadedness, presyncope, syncope, or even cardiac arrest. Additionally, these findings may even result in life-threatening arrhythmias such as supraventricular tachycardia (SVT), atrial fibrillation, and atrial flutter [10]. During pregnancy, the factors that contribute to SVT, such as myocardial ischemia, electrolyte imbalance, and elevated blood sugar levels, progressively increase due to the expansion of blood volume, hormonal changes, and heightened anxiety and tension [11]. Therefore, the management of pre-excitation syndrome during pregnancy, especially its cardiac complications such as tachyarrhythmia, has become a challenge in the clinic [11, 12]. Currently, few studies have conducted data analysis of the pregnancy and delivery success rate in patients with pre-excitation syndrome, and there remains no established consensus guiding peripartum management [5, 13–19]. Pregnancy itself is the primary determinant of arrhythmia burden. Conversely, obstetric complications such as premature rupture of membranes (PROM), preterm birth, small for gestational age (SGA), and fetal distress are intricately associated with maternal cardiac complications [15, 16].

Herein, we analyzed the possible cardiovascular and obstetric complications in 309 pregnancies in 280 women (29 women had two pregnancies in this series) with pre-excitation syndrome who were grouped according to the presence or absence of SVT during pregnancy.

## Methods

### Study design and data source

We retrospectively analyzed the medical records of 127,725 pregnant women hospitalized at the West China Second University Hospital, a tertiary referral center, from June 2011 to October 2021. After analyzing the electrocardiogram results of all patients and documenting consultations with cardiologists, individuals who voluntarily terminated their pregnancies and underwent induced abortions were excluded. As a result, a total of 309 patients were diagnosed with pre-excitation syndrome. The prevalence of pre-excitation syndrome in these pregnant women was 0.24% (309/127,725) in this time period. All the 309 pregnant women with pre-excitation syndrome were categorized into SVT and non-SVT groups based on the presence or absence of SVT during pregnancy, to analyze both cardiac and obstetric complications. This study was approved by the Institutional Review Board of West China Second University Hospital (approval number: 2022-185). Informed consent was obtained from all participants or from the participant’s parents and/or legal guardians if the participant was < 18 years of age. All experimental methods performed in this study were in accordance with relevant guidelines and regulations.

### Electrocardiogram (ECG)

Pregnant women underwent routine ECG examinations during the first trimester of pregnancy in accordance with the hospital’s prenatal examination card requirements, or at 34 weeks of pregnancy if they had not received routine prenatal examinations earlier due to transferring from other hospitals. The 24 h ambulatory ECG was performed on pregnant women who had abnormal ECG patterns or presented with symptoms such as dizziness, weakness, and syncope but without abnormal ECG in early pregnancy. SVT was defined by a narrow complex (QRS < 120 ms) at a rate > 100 beats per min (bpm). This study included atrioventricular nodal re-entrant tachycardia (AVNRT), atrioventricular reciprocating tachycardia (AVRT), and atrial tachycardia. All pregnant women were divided into SVT and non-SVT groups according to the presence or absence of SVT during pregnancy.

### Echocardiography

All the patients with abnormal ECGs underwent echocardiography to determine whether they had other organic heart diseases. Additionally, cardiac ultrasound was employed to exclude the presence of pulmonary hypertension. In the second trimester of pregnancy, all pregnant women received fetal echocardiography and fetal systemic ultrasound to exclude fetal heart disease and other fetal abnormalities.

### Other methods for diagnostic assistance

All participants underwent a clinical examination. Additionally, the following comorbidities were considered in the clinical characterization of the patients: organic heart disease, hypertension, and other pregnancy-associated diseases. Pregnant women who were suspected to have pre-excitation syndrome (according to the medical history, physical examination, ECG, 24 h ambulatory ECG, and echocardiography) or who were previously diagnosed with pre-excitation syndrome were asked to consult a cardiologist. Cardiologists further classified these pregnant women with pre-excitation syndrome into WPW (types A and B) and LGL subtypes. Vascular ultrasound was employed for diagnosing peripheral embolism, as necessary.

### Definitions of terms

The definition of left ventricular systolic dysfunction is characterized by a left ventricular ejection fraction below 40%. The ECG criteria for Left Atrial Enlargement (LAE) were that the ECG produces a broad, bifid P wave in lead II (P mitral) and enlarges the terminal negative portion of the P wave in V1. Preterm labor was defined as cervix opening after week 20 and before week 37 of pregnancy. Small for gestational age (SGA) was defined as a neonate with a birth weight < 10th centile for their gestational age. Fetal distress is an emergency pregnancy, labor, and delivery complication in which a neonate experiences oxygen deprivation (birth asphyxia). Polyhydramnios is defined as an excessive accumulation of amniotic fluid, characterized by a volume exceeding 2000 ml at any stage of pregnancy. It is typically assessed through ultrasound, where the maximum vertical depth of the dark zone in the amniotic fluid measures ≥ 8 cm or the amniotic fluid index (AFI) is ≥ 25 cm. Conversely, oligohydramnios refers to a reduction in the volume of amniotic fluid during late pregnancy. This can be determined through ultrasound by measuring an amniotic fluid depth (AFD) of ≤ 3 cm or an AFI of ≤ 8 cm. The term “fetal macrosomia” is used to describe newborns with a birth weight equal to or greater than 4 kg. And pregnancy with thrombocytopenia (PT) refers to a platelet count below 100 × 10^9^/L.

### Statistical analysis

Descriptive statistical methods were used to represent variables such as frequency, percentage, mean, standard deviation (SD), and range. Normally distributed variables are expressed as the mean ± SD, and the differences between groups were compared using Student’s *t* test. Fisher’s exact probability test or Chi-square test was used for comparing classification variables such as cardiac and obstetric complications. The distribution of blood loss is presented as the median ± interquartile range (IQR) and was compared using Mann–Whitney *U* test. These statistical methods have also been used in our other studies on pregnancy complicated with heart disease [20]. We used SPSS version 25.0 software (IBM Corp, Armonk, NY, USA) for all statistical analysis, and alpha = 0.05 was used as the cutoff for statistical significance.

## Results

### Patient characteristics

A total of 309 pregnant women were recruited in this study involving 280 patients, 29 of whom experienced two pregnancies during the 10 years, with an average age of 30.7 ± 4.5 years (range 17–43 years). In the 309 cases with pre-excitation syndrome during pregnancy, 62 (20.1%, 62/309) had a history of SVT. However, among 127,416 pregnant women without pre-excitation syndrome, 62 cases (0.0485%, 62/127416) had a history of SVT. The average age of pregnant women was 31.1 ± 4.4 years in the SVT group and 30.6 ± 4.6 years in the non-SVT group. In all cases, 62.5% (193/309) were primiparous, among whom, 9.8% (19/193) were primiparity in advanced age (primiparous age > 35 years).

### Cardiac complications

The clinical characteristics and complications of the recruited patients are shown in Table 1. The history of SVT, cardiac function, and complicated heart abnormalities were significantly different between the two groups. Among all 309 patients, 79.9% did not have SVT during pregnancy, but patients with a history of SVT before pregnancy had a significantly higher probability to develop SVT during pregnancy (OR = 12.521, indicating a 12-fold increase in the risk of SVT). Among all patients, 77 (24.9%) had a diagnosis of pre-excitation syndrome prior to pregnancy or in a previous pregnancy. Among this group, 32 patients experienced the manifestation of these conditions during pregnancy and subsequently developed SVT. In the 62 patients with SVT during pregnancy, 22 patients (35.5%) had a history of SVT. All patients with a history of SVT, regardless of whether they were treated with drugs or untreated, experienced SVT during pregnancy. However, among the 11 patients who underwent radiofrequency ablation (RFCA) before pregnancy, seven did not have SVT during pregnancy and exhibited good cardiac function. Additionally, it was found that cardiac function during pregnancy was significantly different between the SVT and non-SVT groups. Among the 247 patients without SVT, 244 (98.8%) had a cardiac function grade (NYHA-FC, the New York Heart Association) of I–II. Most patients (295/309, 95.5%) did not develop organic lesions; however, some patients had aortic regurgitation (AR), mitral regurgitation (MR), tricuspid regurgitation (TR), atrial septal defect (ASD), hypertrophic cardiomyopathy (HCM), LAE, and pericardial effusion, all of which increased the risk of SVT during pregnancy.

**Table 1:** Clinical characteristics and cardiac complications in patients with pre-excitation syndrome (*N* = 309)

Parameters	No	SVT during pregnancy (*N* = 62, %)	None SVT during pregnancy (*N* = 247, %)	*P*-value	OR (95% CI)
The time of diagnosis					
Before pregnancy	77	32 (51.6)	45 (18.2)	0.000	4.788 (2.644–8.670)
During pregnancy	232	30 (48.4)	202 (81.8)		
None SVT history	280	40 (64.5)	240 (97.2)	0.000	12.521 (5.605–27.968)
SVT history before pregnancy	29	22 (35.5)	7 (2.8)		
Radiofrequency ablation	11	4 (6.45)	7 (2.8)	0.024	
Medical treatment	12	12 (19.35)	0	0.000*	
Untreated	6	6 (9.7)	0	0.000*	
Pre-excitation syndrome subtypes					14.608 (4.202–50.775)
WPW	255	50 (80.6)	205 (83.0)	0.709	
LGL	54	12 (19.4)	42 (17.0)		
NYHA-FC					
I—II	295	51 (82.3)	244 (98.8)	0.000	
III—IV	14	11 (17.7)	3 (1.2)		
Left ventricular systolic function					
Normal	307	60 (96.8)	247 (100)	0.040*	
Dysfunction	2	2 (3.2)	0		
With other arrhythmias					7.968 (2.825–22.470)
None†	232	45 (72.6) /17	187 (75.7)/60	0.624	
Sinus arrhythmia	27	7 (11.3)	20 (8.1)	0.451	
APB	23	5 (8.1)	18 (7.3)	1.000	
VPB	11	2 (3.2)	9 (3.6)	1.000*	
APB + VPB	16	3 (4.8)	13 (5.3)	1.000*	
With heart abnormalities					
None†	295	52 (83.9)	242 (98.0)	0.000*	
AR	1	0	1 (0.4)	1.000*	
MR	2	0	2 (0.8)	1.000*	
TR	4	4 (6.5)	1 (0.4)	0.006*	
ASD	2	1 (1.6)	1 (0.4)	0.362*	
HCM	1	1 (1.6)	0	0.201*	
Left atrial enlargement	2	2 (3.2)	0	0.040*	
Pericardial effusion	2	2 (3.2)	0	0.040*	

The total number of cases were 309 involving 280 patients, 29 of whom experienced two pregnancies during the 10 years. The patients with atrial septal defect all had undergone surgery before pregancy

*N*/*n* number, *SVT* supraventricular tachycardia, *WPW* Wolff–Parkinson–White syndrome, *LGL* Lown-Ganong-Levine syndrome, *NYHA-FC* cardiac function grading (New York Heart Association), *APB* atrial premature beat, *VPB* ventricular premature beats, *AR* aortic regurgitation, *MR* mitral regurgitation, *TR* tricuspid regurgitation, *ASD* atrial septal defect, *HCM* hypertrophic cardiomyopathy

^†^None: indicated that this situation has not yet arisen; * Fisher exact probability method was used

### Obstetric complications

The clinical data of obstetric complications, including maternal and fetal complications, are summarized in the upper part of Table 2. The maternal complications included premature rupture of membranes (PROM), placental abruption, gestational diabetes mellitus (GDM), hypothyroidism, pre-eclampsia, monochorionic diamnionic (MCDA) twin gestations, intrahepatic cholestasis of pregnancy (ICP), thrombocytopenia, thalassemia, and umbilical cord prolapse. The most common maternal complication was PROM (65/309, 21.04%), followed by GDM (64/309, 20.71%). The prevalence of GDM in the SVT group was significantly higher than that in the non-SVT group, and the risk in SVT group increased by twofold (OR = 2.197).

**Table 2:** Pregnancy outcomes according to maternal cardiac anomaly

Outcomes of pregnancies	No. (%) (*N* = 309/*F* = 319)†	SVT during pregnancy (*N* = 62/*F* = 65)	None SVT during pregnancy (*N* = 247/*F* = 254)	*P*-value	OR (95% CI)
Fetal complications					
Prematurity#	30 (9.4)	8 (12.31)	22 (8.66)	0.369	–
SGA	3 (0.94)	1 (1.54)	2 (0.79)	0.496*	–
Fetal distress	11 (3.45)	1 (1.54)	10 (3.94)	0.472*	–
Polyhydramnios	5 (1.57)	0	5 (1.97)	0.587*	–
Oligohydramnios	2 (0.63)	1 (1.54)	1 (0.39)	0.367*	–
Fetal macrosomia	2 (0.63)	0	2 (0.79)	1.000*	–
Fetal cardiac abnormalities					
Fetal single ventricle	1 (0.31)	0	1 (0.39)	1.000*	
Fetal VSD	1 (0.31)	1 (1.54)	0	0.204*	
Maternal complications					
PROM	65 (21.04)	11 (17.74)	54 (21.86)	0.495	–
Placental abruption	5 (1.62)	2 (3.23)	3 (1.21)	0.263*	2.197 (1.177 ~ 4.102)
GDM	64 (20.71)	20 (32.26)	44 (17.81)	0.015	
Hypothyroidism	17 (5.5)	3 (4.84)	14 (5.67)	1.000*	
Preeclampsia	4 (1.29)	1 (1.61)	3 (1.21)	1.000*	
MCDA	10 (3.24)	3 (4.84)	7 (2.83)	0.426*	
ICP	16 (5.18)	6 (9.68)	10 (4.05)	0.102	–
Thrombocytopenia	11 (3.56)	2 (3.23)	9 (3.64)	1.000*	4.272 (1.865 ~ 9.785)
Thalassemia	7 (2.27)	2 (3.23)	5 (2.02)	0.631*	
Umbilical cord prolapse	1 (0.32)	1 (1.61)	0	0.201*	
Mode of delivery					
Cesarean section	215 (69.58)	55 (88.7)	160 (64.78)	0.000	
Vaginal delivery	90 (29.13)	7 (11.3)	83 (33.6)		
Assisted vaginal delivery	4 (1.29)	0	4 (1.62)		
Transfer to ICU	5 (1.62)	5 (8.06)	0	0.000*	–
Transfer to neonatology	22 (6.9)	7 (10.77)	15 (5.91)	0.292	–
IUFD	4 (1.25)	3 (4.62)	1 (0.39)	0.028*	–
Postpartum hemorrhage	12 (3.88)	5 (8.06)	7 (2.83)	0.001*	
Blood loss at delivery (ml)		359.22 ± 130.88	339.78 ± 201.11	0.470	
Mean neonatal weight (kg)		3117.65 ± 629.97	3171.12 ± 535.69	0.492	
HOD (day)		5.935 ± 4.731	4.238 ± 2.562	0.000	

*N* number, *F* fetal, *SVT* supraventricular tachycardia, *SGA* small for gestational age, *PROM* premature rupture of membranes, *GDM* gestational diabetes mellitus, *MCDA* monochorionic diamnionic twin, *ICP* intrahepatic cholestasis of pregnancy, *VSD* ventricular septal defect, *ICU* intensive care unit, *IUFD* Intrauterine fetal demise, *HOD* Hospitalization days (total days in hospital)

^†^The total number of fetuses was 319 because there are 10 patients with twin pregnancies (maternal number = 309); #Prematurity: preterm labor, was defined as cervix opening after week 20 and before week 37 of pregnancy

^*^Fisher exact probability method was used

Since 10 cases were complicated with MCDA twin gestations, 319 fetuses were observed in 309 cases. Fetal complications, including prematurity, SGA, fetal distress, polyhydramnios, oligohydramnios, and fetal macrosomia, were reviewed. However, there was no significant difference in fetal complications between the two groups.

### Maternal and perinatal outcomes

Among all recruited patients, 215 (69.58%) patients underwent cesarean section, 90 (29.13%) patients had a vaginal delivery, and four (1.29%) patients underwent an assisted vaginal delivery. The cesarean section rate was 88.7% (55/62) in the SVT group, which was significantly higher than that in the non-SVT group (64.78%, 160/247).

No cases of maternal death were observed, but five patients were transferred to the intensive care unit (ICU) after delivery because of heart failure with arrhythmia in the perinatal period. As shown in the middle and lower parts of Table 2, fetal cardiac abnormalities were observed in two patients (2/319, 0.63%), with no statistical significance between the two groups. Moreover, there was no significant difference in blood loss during delivery and mean newborn weight between the two groups. However, postpartum hemorrhage, intrauterine fetal demise (IUFD), and hospital stay (HOD) were significantly different (*P* < 0.05). Postpartum hemorrhage accounted for 8.06% (5/62) of patients with SVT during pregnancy, while 2.83% (7/247) of patients without SVT during pregnancy. The incidence of intrauterine fetal demise was 4.62% (3/65) among pregnant patients with SVT, compared to only 0.39% (1/254) among those without SVT during pregnancy; And duration of hospital stay was significantly prolonged in patients who experienced episodes of supraventricular tachycardia (SVT) during pregnancy, with an average length of 5.935 ± 4.731 days, whereas the duration of hospitalization without SVT episodes during pregnancy was 4.238 ± 2.562 days. Twenty-two neonates were transferred to Neonatal Intensive Care Unit, and no neonatal deaths occurred, but there were four fetal deaths (three cases in the SVT group and one case in the non-SVT group). In the SVT group, one patient with SVT in the third trimester of pregnancy developed placental abruption approximately 30 min later, resulting in IUFD, and two patients with multiple occurrences of SVT were induced in the second trimester after IUFD. One patient in the non-SVT group was induced at 15 weeks due to the fetal lymphatic sac, which can be caused by various factors such as infections, metabolic abnormalities, ischemia, chromosomal abnormalities or drug effects.

### Two pregnancy outcomes of one woman

In this study, 29 women experienced two pregnancies in 10 years, among whom, 22 (75.9%) women were diagnosed with pre-excitation syndrome in the first pregnancy, and seven (31.8%) women developed SVT during pregnancy; five of them received RFCA at the Department of Cardiology after the first delivery, and they did not have SVT in the second pregnancy or delivery (Table 3).

**Table 3:** Different outcomes of the same patient in two pregnancies

Total number = 29^#^	First delivery		Second delivery	
	Diagnosis before pregnancy	Diagnosis during pregnancy	After RFCA	Untreated
Number (%)	7 (24.1)	22 (75.9)	5 (17.2)	24 (82.8)
SVT during pregnancy	2 (28.6)	7 (31.8)	0	5 (20.8)
None SVT during pregnancy	5 (71.4)	15 (68.2)	5 (100)	19 (79.2)
*P*-value	1.000*		0.002*	
NYHA-FC I–II	6 (85.7)	21 (95.5)	5(100)	22 (91.7)
NYHA-FC III–IV	1 (14.3)	1 (4.5)	0	2 (8.3)
*P*-value	0.431*		1.000*	
Transfer to ICU	0	0	0	0
Transfer to neonatology	0	2 (9.1)	0	1 (4.17)
Neonatal death	0	0	0	1 (4.17)

*RFCA* radiofrequency ablation, *SVT* supraventricular tachycardia, *NYHA-FC* cardiac function grading (New York Heart Association)

^#^The 29 patients experienced two pregnancies during 10 years

*Fisher exact probability method was used

## Discussion

Pre-excitation syndrome is a collective term that refers to premature ventricular activation through accessory or anomalous conduction pathways [1–9]. WPW, a classic subtype of pre-excitation, is characterized by the presence of a short PR interval and a broad QRS with a delta wave on ECG, whereas the LGL subtype only shows a short PR interval and a normal QRS complex on ECG [5–8, 21, 22]. Ventricular pre-excitation can predispose pregnant women to re-entry arrhythmias, with clinical manifestations of pre-excitation, paroxysmal tachycardia, and related symptoms, including palpitations, dizziness, and/or syncope [6, 21, 22].

In large-scale general population studies involving children and adults, the prevalence of WPW is estimated as 1–3 per 1000, and the incidence of WPW among first-degree relatives is 5.5 per 1000 [22]. Approximately 65% of adolescents and 40% of individuals > 30 years old with a WPW pattern on resting ECG have no symptoms [23]. Consequently, it is difficult to identify asymptomatic patients with pre-excitation syndromes. In this study, 75.1% of patients were diagnosed with pre-excitation syndrome by ECG examination in the second or third trimester of pregnancy, and most of them had no symptoms.

However, the increase in blood volume and changes in hormone levels predispose pregnant women with pre-excitation syndrome to SVT, leading to an increased risk of adverse events [6, 19]. The treatment of serious arrhythmia caused by pre-excitation syndrome during pregnancy is more difficult compared to that in nonpregnancy, and there is currently no clear treatment specification. At present, vagal maneuvers and intravenous injection of adenosine are recommended as Class I therapies for managing SVT [23]. If the patient with pre-excitation is unwilling to undergo ablation, medications such as flecainide and propafenone can be used as Class IIA recommendations (in the absence of structural heart disease). If the patient is hemodynamically unstable, synchronous cardioversion is recommended. Nevertheless, none of these effective treatments can be used in pregnancy owing to the risk of fetal exposure to ionizing radiation and fetal arrhythmia [24, 25]. Due to limited treatment options, the management of SVT during pregnancy remains a thorny clinical challenge [24]. In this study, none of the recruited cases with SVT received RFCA during pregnancy. Cardiologists suggest that these patients should undergo RFCA after delivery, especially those who wish to have another child. Accordingly, we focused on 29 patients who experienced two pregnancies during our study period. Among them, five patients received RFCA after the first delivery and obtained better outcomes in the second pregnancy. However, RFCA was performed in only five patients, which is insufficient to draw conclusions. Future research with a larger sample size is required to further explore these hypotheses.

We found that a history of SVT before pregnancy, cardiac function, and heart organic abnormalities was related to the occurrence of SVT during pregnancy. Although only 24.9% of all patients were diagnosed with pre-excitation syndrome before pregnancy or in the previous pregnancy, 51.6% of them experienced SVT during pregnancy. We speculate that this may be related to imprecise or no treatment in these cases (11 of them received RFCA before pregnancy) even if some had previously experienced recurrent SVT. Additionally, RFCA as an effective treatment for pre-excitation syndrome has a certain failure rate. Indeed, it has been reported that the success rate of the first RFCA is 91.2%, while that of the second ablation is 80% [26]. In this study, one patient underwent two RFCA procedures, but SVT occurred again during pregnancy. Overall, 95.5% of patients had a cardiac function grade of I–II, and few patients developed serious cardiac complications. All patients with left ventricular systolic dysfunction and most patients with a cardiac function grade of III–IV during pregnancy were from the SVT group.

A recent study reported that a young patient with WPW syndrome and postpartum cardiomyopathy (PPCM) developed symptomatic arrhythmias (tachycardia), suggesting that WPW syndrome is a pre-excitation/accessory pathway-induced arrhythmia related to pregnancy and can occur along with PPCM [5]. Among all patients included in this study, one patient with hypertrophic cardiomyopathy developed SVT in the third trimester and was transferred to the ICU after cesarean section.

Previous literature has shown that patients with pre-excitation abnormalities are at a high risk of life-threatening perioperative arrhythmias [3, 27]. SVT can present at any stage of pregnancy, including labor. The etiology of SVT during labor is multifactorial. Increased cardiac output stretches the myocardial tissues, predisposing pregnant women to tachyarrhythmia. Moreover, catecholamine release, electrolyte disturbances such as hyperkalemia during labor, and vasopressors administered to treat post-epidural hypotension are other common triggers of SVT during labor [27, 28]. Based on this, cesarean delivery may be relatively safe for patients with pre-excitation syndrome, especially those with a history of SVT, because of the higher catecholamine release during vaginal delivery [16, 20, 29]. However, anesthesiologists believe that spinal anesthesia during cesarean section can result in a series of blood pressure changes. It is difficult to maintain blood pressure within the reference range in the absence of vasopressors, but vasopressors may prompt the onset of SVT. Therefore, the delivery mode for pregnant women with pre-excitation syndrome is challenging. We believe that the delivery mode for patients with SVT history should be determined prudently, but SVT is not an absolute indication of cesarean section. Multidisciplinary team assessments are necessary to achieve the best benefits for both mothers and neonates, and vaginal delivery is also possible while ensuring the safety of mothers and neonates [15, 16]. In our study, 30.42% of patients with vaginal delivery or assisted vaginal delivery achieved good outcomes.

Some obstetric complications were observed, including GDM, PROM, SGA, pre-eclampsia, and fetal distress. Five patients with critical perinatal conditions were transferred to the ICU, and no cases of maternal death occurred. All patients complicated with heart disease were managed conservatively by a multidisciplinary team. Most mothers and newborns had good outcomes, except for four fetuses that died in the second and third trimesters. However, given the recognized familial clustering of many SVT etiologies [22], the potential for fetal SVT should also be expected, and it is important to pay more attention to its associated complications, including fetal arrhythmia, in utero heart failure and death. It is necessary of routine fetal echocardiographic examinations for all fetuses during the mid to late stages of pregnancy, as well as enhancing fetal heart monitoring throughout this period. In case any abnormalities are detected, it is advisable to promptly conduct a follow-up fetal echocardiography examination.

As mentioned above, pregnant women are prone to develop new-onset or recurrent arrhythmias as a result of physiological changes during pregnancy. Pregnancy creates an arrhythmogenic environment, leading to an increased risk of arrhythmia in the pregnant population [15]. Our results showed that pregnant women with GDM (glucose intolerance diagnosed during pregnancy) were more likely to experience SVT during pregnancy. Previous literature has reported that the deterioration of the cardiac autonomic nervous system in patients with diabetes is associated with an increase in cardiac and arrhythmogenic mortality [17]. Emerging evidence has also demonstrated that diabetes affects the electrical conduction system of the heart, culminating in lethal arrhythmia and sudden cardiac death. Hypoglycemia, hyperglycemia, and glucose fluctuations can induce arrhythmia by activating various pathways [18, 30]. Therefore, more attention should be paid to the relationship between diabetes and arrhythmia.

The limitations of our work as follows: this is a retrospective analysis so we were limited to know more information about the subtypes of pre-excitation syndrome and the metabolic diseases other than diabetes. In addition, we did not clear whether pregnancy itself is a factor that promotes preexcitation, or whether these patients suffered from concealed pre-excitation syndrome before pregnancy that have not been found by us. So we need more perfect pre-pregnancy examination program which should include cardiac ultrasound and electrocardiogram. We advocate for greater consideration of suggestions and assistance from cardiologists and anesthesiologists in future studies, as this would enable us to develop more comprehensive plans regarding the mode of delivery and individualized treatment for pre-excitation syndrome during pregnancy.

Despite the limitations, the strength of the study is that this study determined the correlation between supraventricular tachycardia and adverse maternal and fetal outcomes in patients with pre-excitation syndrome, and founded the risk factors leading to supraventricular tachycardia during pregnancy.

## Conclusion

In summary, SVT as a life-threatening arrhythmia during pregnancy, is closely related to poor outcomes in patients with pre-excitation syndrome. A multidisciplinary approach is crucial to balance maternal and obstetrical outcomes, especially for pregnant women with a history of SVT before pregnancy.

## Data Availability

All data and materials support our claims and comply with field standards.

## Code availability

All software applications support our claims and comply with field standards.
